# Cold snaring biopsies to increase screening efficacy during endoscopic surveillance of patients at high risk of diffuse gastric cancer

**DOI:** 10.1055/a-2262-7988

**Published:** 2024-03-01

**Authors:** Marie Lemoine Laroussinie, Mathieu Pioche, Laura Calavas, Tanguy Fenouil, Jean-Christophe Saurin, Arnaud Pasquer, Nicolas Benech

**Affiliations:** 1 Gastroenterology Unit, Edouard Herriot Hospital, Hospices Civils de Lyon, Lyon, France; 2 Anatomopathology Unit, Hospices Civils de Lyon, Lyon, France; 3 Surgery Unit, Edouard Herriot Hospital, Hospices Civils de Lyon, Lyon, France; 4 Hepatogastroenterology Unit, Hôpital de la Croix-Rousse, Hospices Civils de Lyon, Lyon, France

Diffuse gastric cancer (DGC) is a poorly differentiated adenocarcinoma of the stomach characterized by independent “signet-ring” cells that invade the gastric wall with mainly submucosal infiltration resulting in delayed diagnosis 1 . Mutations in the *CDH1* gene, encoding E-cadherin, have been identified in families with multiple cases of gastric cancer, with a 50%–80% increased risk of DGC in carriers 2 . In cases with a proven constitutional *CDH1* mutation, guidelines recommend prophylactic total gastrectomy between the ages of 20 and 30 years, or endoscopic surveillance if surgery is refused or postponed 3 .

Given the multiple possible localizations and frequency of small carcinomatous foci, a minimum of 30 biopsies divided into six zones using standard forceps is recommended 3 4 . However, multiple sampling of the gastric mucosa using conventional biopsy forceps still represents a limited surface area that can be analyzed. Increasing the size of the biopsy sample using cold snaring may increase diagnostic sensitivity.

We report here the case of a 33-year-old patient with regular endoscopic surveillance after identification of a *CDH1* mutation in a familial context.

Gastroscopy and endoscopic ultrasound were performed, with no findings of macroscopic abnormalities, parietal thickening, or suspicious adenopathy ( Video 1 ). To increase the size of the tissue sampled for histological analysis, 24 cold snaring biopsies (6 upper fundus, 6 body, 6 lower body, and 6 antrum) were performed. Pathological analysis revealed a single focal area of independent cell adenocarcinoma in the chorion (pTis) seen only in one sample. A gastrectomy was then performed, with a millimetric focal adenocarcinoma with signet-ring cells on the surgical specimens.

Gastric sampling using random cold snaring of the whole stomach with visualization of the corresponding resected area.Video 1

Histopathological analysis was improved, with the average size of biopsy fragments being significantly larger with cold snaring than with standard forceps, and with a reduction in crush artifacts ( Fig. 1 ).

**Fig. 1:**
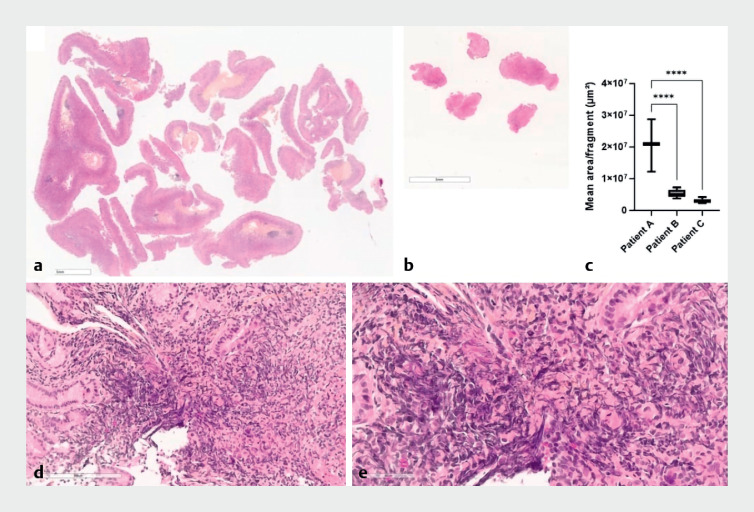
Histopathological characterization (hematoxylin and eosin) of specimens from patients followed endoscopically due to familial predisposition for *CDH1* mutation-related gastric adenocarcinoma. **a** Cold snaring biopsy. **b** Standard forceps biopsy. **c** The mean size of biopsy fragment was significantly increased with cold snaring (patient A) compared with standard forceps (patients B and C), based on the examination of three patients with 18, 30, and 28 fragments biopsied, respectively (one-way analysis of variance with Dunnett’s multiple comparisons, **** *P* < 0.0001; GraphPad Prism v10.0.0). **d** Cold snaring sample: the crushing artifacts often observed with standard forceps were not seen in the cold snaring biopsies (×20 magnification; **e** ×40 magnification).

In conclusion, this case suggests that large samples obtained with cold snaring could potentially decrease the focal adenocarcinoma miss rate in *CDH1* mutation carriers.

Endoscopy_UCTN_Code_TTT_1AO_2AC

## Notes

Endoscopy E-Videos https://eref.thieme.de/e-videos
*E-Videos* is an open access online section of the journal *Endoscopy* , reporting on interesting cases and new techniques in gastroenterological endoscopy. All papers include a high-quality video and are published with a Creative Commons CC-BY license. Endoscopy E-Videos qualify for HINARI discounts and waivers and eligibility is automatically checked during the submission process. We grant 100% waivers to articles whose corresponding authors are based in Group A countries and 50% waivers to those who are based in Group B countries as classified by Research4Life (see: https://www.research4life.org/access/eligibility/ ). This section has its own submission website at https://mc.manuscriptcentral.com/e-videos .

## References

[1] Ikoma N Agnes A Chen H-C Linitis plastica: a distinct type of gastric cancer J Gastrointest Surg 2020 24 1018 1025 10.1007/s11605-019-04422-7 31754987

[2] Hansford S Kaurah P Li-Chang H Hereditary diffuse gastric cancer syndrome: CDH1 mutations and beyond JAMA Oncol 2015 1 23 32 10.1001/jamaoncol.2014.168 26182300

[3] Fitzgerald RC Hardwick R Huntsman D Hereditary diffuse gastric cancer: updated consensus guidelines for clinical management and directions for future research J Med Genet 2010 47 436 444 10.1136/jmg.2009.074237 20591882 PMC2991043

[4] van der Post RS Vogelaar IP Carneiro F Hereditary diffuse gastric cancer: updated clinical guidelines with an emphasis on germline CDH1 mutation carriers J Med Genet 2015 52 361 374 25979631 10.1136/jmedgenet-2015-103094 PMC4453626

